# Secular trends in HIV/AIDS mortality in China from 1990 to 2016: Gender disparities

**DOI:** 10.1371/journal.pone.0219689

**Published:** 2019-07-18

**Authors:** Disi Gao, Zhiyong Zou, Bin Dong, Wenjing Zhang, Tianqi Chen, Wenxin Cui, Yinghua Ma

**Affiliations:** Institute of Child and Adolescent Health, School of Public Health, Peking University Health Science Center, Beijing, China; University of South Carolina Arnold School of Public Health, UNITED STATES

## Abstract

**Objectives:**

HIV/AIDS has become the leading cause of death by infectious disease in China since 2009. However, the trend of gender disparities in HIV/AIDS has not been reported in China since 1990. Our study aimed to explore the secular trend of HIV/AIDS mortality in China from 1990 to 2016 and to identify its gender disparities over the past 27 years.

**Method:**

The mortality data of HIV/AIDS were obtained from the Global Burden of Disease Study 2016 (GBD 2016). Logistic regression was used to estimate the prevalence odds ratio (POR) of gender for HIV/AIDS mortality in different surveys.

**Results:**

The standardized mortality of HIV/AIDS in China rose dramatically from 0.33 per 100,000 people in 1990 to 2.50 per 100,000 people in 2016. The rate of HIV/AIDS mortality increased more quickly in men than in women, and the sex gap of mortality of HIV/AIDS widened. By 2016, the HIV/AIDS mortality in men was 3 times that in women and was 5.74 times that in women within the 75- to 79-year-old age group.

**Conclusions:**

The mortality of HIV/AIDS in China is increasing, with a widening gender disparity. It is critical for policymakers to develop policies to eliminate these disparities and to ensure that everyone can live a long life in full health.

## Introduction

Human immunodeficiency virus (HIV) and the consequent acquired immunodeficiency syndrome (AIDS) have caused a globally devastating pandemic, which led to major distortions in population age-sex distributions in the most affected areas[1, 2]. Worldwide, 77.3 million people have become infected with HIV, and 35.4 million people died from AIDS-related illnesses by 2018. The number of global deaths caused by HIV/AIDS reached a peak of nearly 1.9 million in 2006 and declined afterwards. In 2017, the number of AIDS-related deaths was nearly 1 million, which had dropped 51% compared with 2006[3]. In the northeast of South Africa, more than 60% of the deaths that occurred between 1992 and 2013 could be attributed to HIV. Although there was a 30% decrease in adult HIV-related mortality between 2007 and 2008, most South African villages still experienced a small increase in mortality from 2007 to 2013[4]. In some Eastern Mediterranean Regions (EMRs), the male mortality rate of HIV/AIDS increased from 0.38 per 100,000 to 2.15 per 100,000, with an increase of 6.7% annually since 1990, which was double that among females[5].

In China, the HIV epidemic has continued to expand, and the mortality attributable to HIV/AIDS has become a major public health concern[6]. In July 2017, approximately 728,270 persons were living with HIV/AIDS in China, and 223,798 died from AIDS-related illnesses[7]. Since HIV/AIDS has become the leading infectious cause of death in 2009[8], the number of reported HIV cases has increased over time, and the number of deaths related to HIV/AIDS has also increased from 5,544 in 2007 to 15,251 in 2017[9].

Although HIV/AIDS mortality in both males and females has rapidly increased in different areas of the world, gender differences are apparent. In most countries, men have higher mortality than women. The results of studies from South Africa and Zimbabwe have shown higher mortality among males than among females[10, 11]. In other countries, such as Malawi and Tanzania, females have had a significantly higher survival rate than similarly situated males[12, 13]. A 2-year follow-up study in China also suggested that women had an overall lower mortality[14]. However, there are no reports on how gender disparities in HIV/AIDS have changed over time in Chinese individuals. Therefore, a better understanding of these gender differences in HIV/AIDS is critical for AIDS prevention and control in China.

In this study, we used data from the Global Burden of Disease Study 2016 (GBD 2016) to identify the trend in gender differences over the past 27 years in China. Furthermore, since no study [15–19] has used the GBD 2016 dataset to conduct studies related to HIV/AIDS, we also hope to fill this research gap by using these data.

## Data sources and methods

### Data sources

Data were extracted from the GBD 2016, a large international cooperation project that globally, regionally, and nationally provides age-sex mortality for 264 causes of death from 1980 to 2016, including HIV/AIDS[20]. Original data, which the GBD adapted to estimate the HIV/AIDS mortality, were mainly from the Disease Surveillance Point (DSP) and the Notifiable Infectious Disease Reporting (NIDR) system. Both systems were administered by the Chinese Centers for Disease Control and Prevention[21].

In this study, we extracted and used 2 indicators to analyze HIV/AIDS mortality trends for 14 age groups (10–14, 15–19, 20–24, 25–29, 30–34, 35–39, 40–44, 45–49, 50–54, 55–59, 60–64, 65–69, 70–74, and 75–79) from 1990 to 2016 in China: the age- and sex-specific number of deaths, mortality and their UI (uncertainty interval). The UI was the yield with 95% uncertainty intervals for the estimated data that included all age-specific mortality rates by the Estimation and Projection Package (EPP)-Spectrum model[21]. By using the number of deaths in each age group divided by the mortality rate in each age group, we estimated the population of each age group in China from 1990 to 2016 (see S1 Table).

### Statistical analysis

We calculated the crude death rate of HIV/AIDS (per 100,000 people) among people aged 10–79 in China from 1990 to 2016 by using the numbers of deaths from individuals 10 to 79 years old divided by the estimated total population of individuals 10 to 79 years old; we also used the 2010 national census to determine the standardized mortality. By calculating the ratio of HIV/AIDS mortality of males to females (M/F) for different years for each age subgroup, we were able to calculate the Relative Risk (RR) to assess the gender and age differences at different time points. Data extraction was conducted using SPSS (SPSS 22.0 for Windows, IBM Inc, Chicago, IL, USA), and other analyses were conducted using Excel software, version 2016 (Microsoft, Redmond, Washington).

## Results

### Gender differences in HIV/AIDS mortality trends in China

**Fig 1:**
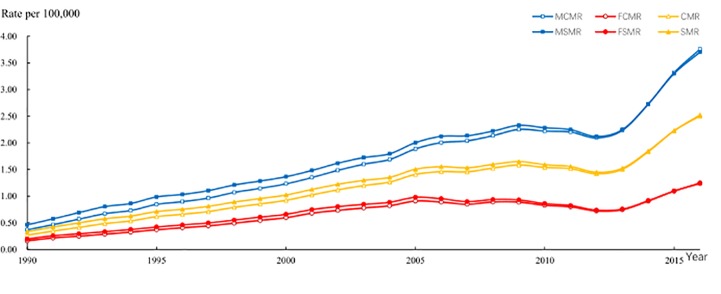
CDR and SMR trends in HIV/AIDS in the 10- to 79-year-old age group from 1990 to 2016. The standardized mortality rate (SMR) of the 10- to 79-year-old age group was based on the distribution of males and females in the 2010 census data.

HIV/AIDS mortality was increasing continuously over the past 27 years. Fig 1 shows the crude death rate (CDR) and the standardized mortality rate (SMR) of HIV/AIDS in the 10- to 79-year-old age group from 1990 to 2016, and HIV/AIDS mortality increased rapidly in both males and females. The male HIV/AIDS standardized mortality increased from 0.46 per 100,000 people in 1990 to 3.70 per 100,000 people in 2016, and the standardized mortality of females increased from 0.20 per 100,000 people in 1990 to 1.24 per 100,000 people in 2016.

### Gender differences in the HIV/AIDS mortality trends by age group

**Fig 2:**
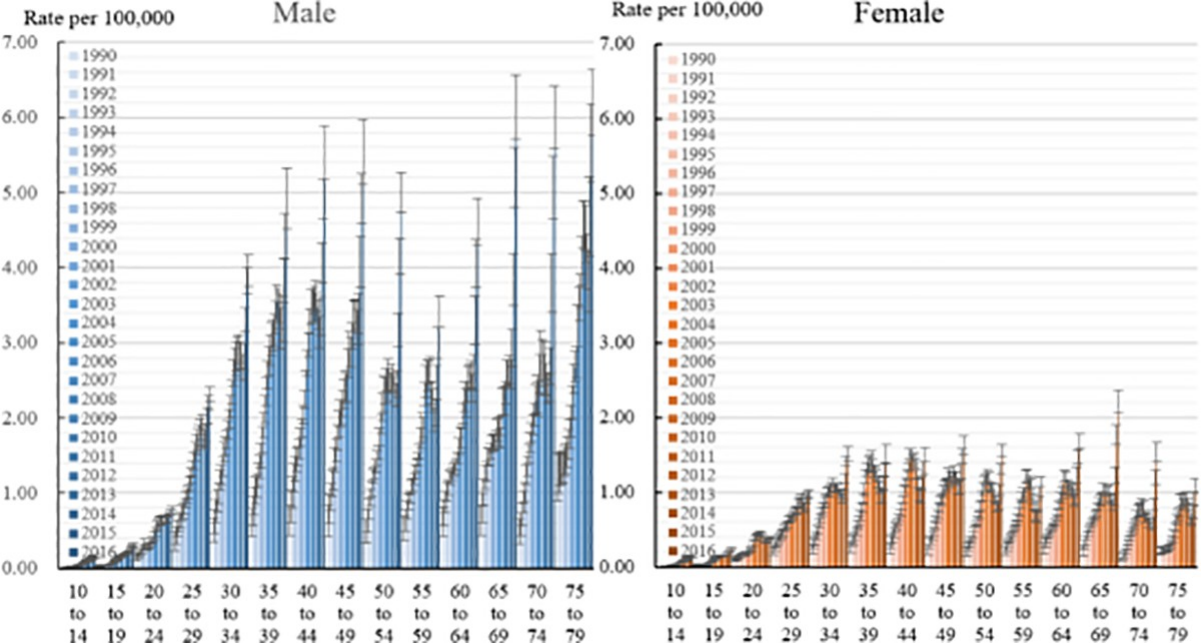
**Age-specific mortality of HIV/AIDS and 95% uncertainty interval (UI) among males (left figure) and females (right figure) aged 10 to 79 years old from 1990 to 2016.** Increasing trends were observed in both males and females, but the increase among females was less than that among males.

Fig 2 shows the trends in HIV/AIDS mortality by age and gender. As shown in Fig 2, the HIV/AIDS mortality for males remained very low in the 10- to 14-year-old age group and in the 15- to 19-year-old age group and then rose significantly until age 49, reaching its highest point of 5.55 per 100,000 in the 40- to 44-year-old age group in 2016; after that, it experienced a slight drop and soon climbed to its second peak again in the 75- to 79-year-old age group of 6.21 per 100,000 deaths in 2016. Compared with males, female mortality trends changed relatively steadily. After a relatively stable increase from age 10 to age 34, when the female HIV/AIDS mortality reached 1.53 per 100,000 in the 30- to 34-year-old age group in 2016, the female HIV/AIDS mortality rate maintained a small fluctuation. During the past 27 years, the mortality of HIV/AIDS in all age subgroups showed an increasing trend for both males and females. However, the increasing trends among females were lower than those among males.

**Fig 3:**
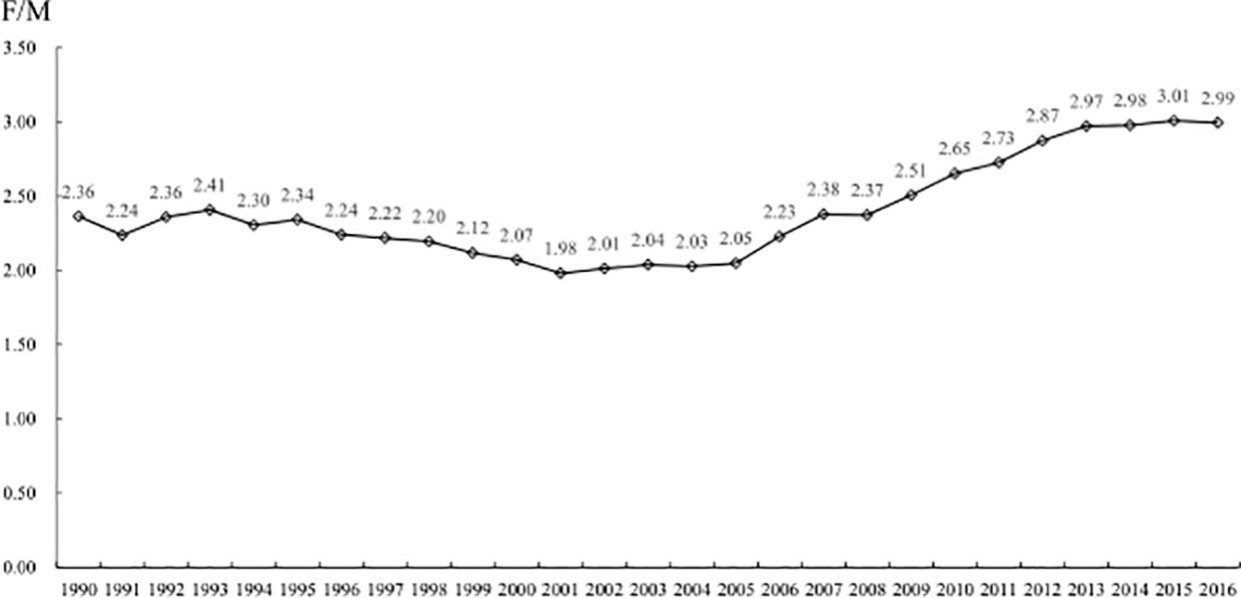
Changes in the ratio of HIV/AIDS mortality of males to females. From 2001 to 2016, the gap between male and female standardized mortality increased significantly.

Fig 3 shows the changes in the ratio of HIV/AIDS mortality between males and females. In 1990, the male HIV/AIDS standardized mortality was 2.36 times that of the female rate. However, since 2001, the gap between male and female standardized mortality increased significantly, from 1.98 times in 2001 to 2.99 times in 2016.

### Age-specific HIV/AIDS mortality risk ratios of males versus females

**Fig 4:**
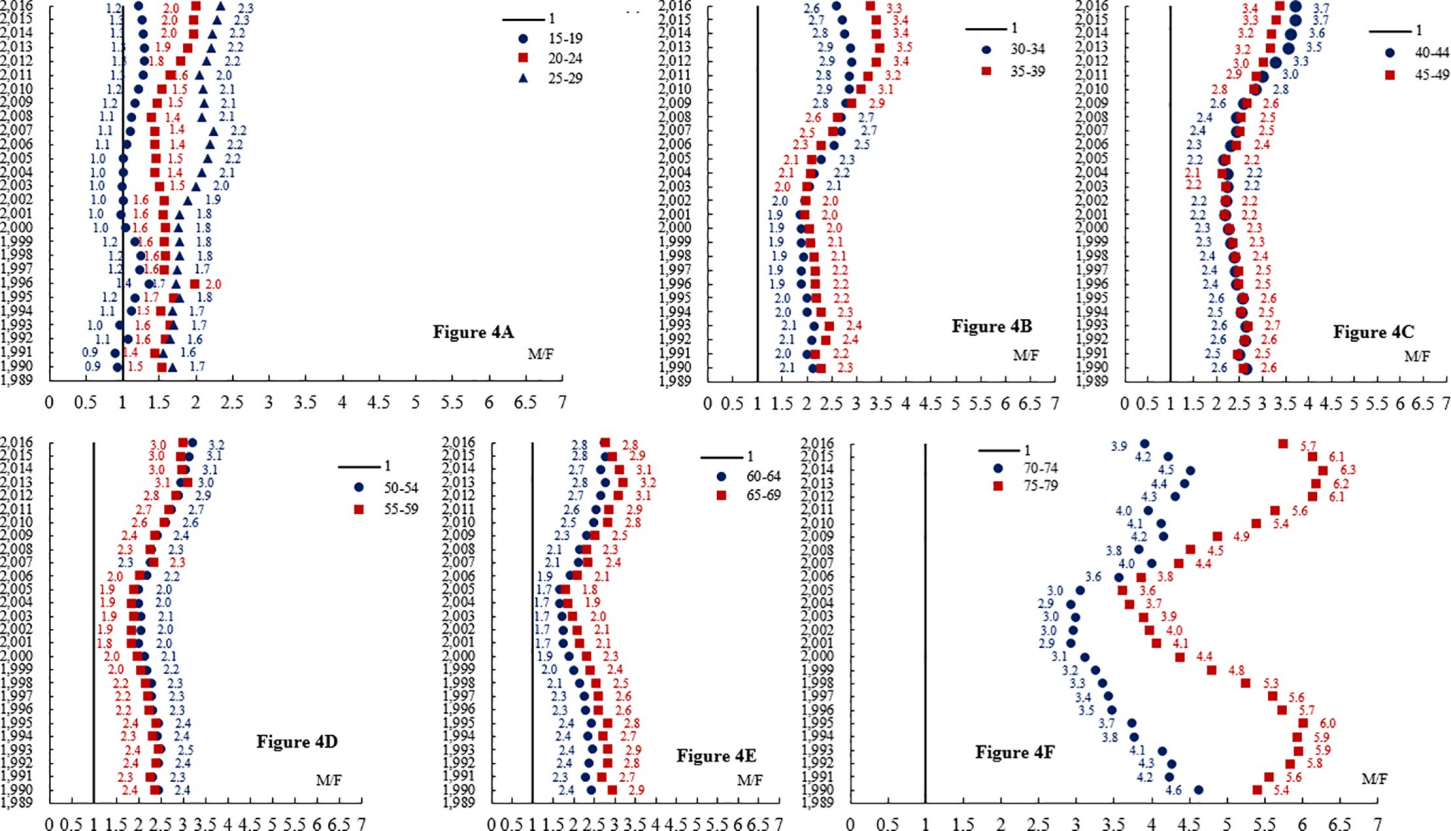
Age-specific HIV/AIDS mortality risk ratios (RRs) of males compared with females in different years in China. The gender difference in HIV/AIDS mortality was readily apparent and increased gradually with age.

As shown in Fig 3, from 1990 to 2016, a significant increasing trend in HIV/AIDS mortality was more obvious in males than in females. We calculated the risk ratio (RR) of male HIV/AIDS mortality versus that of female HIV/AIDS mortality for different years for each age subgroup (Fig 4). In Fig 4A, in the 15- to 19-year-old subgroup, few differences in gender disparities were evident in the past 27 years. For the 20- to 24-year-old subgroup, the gender difference reached its peak in 2016 (RR = 2.0). Similarly, the difference in HIV/AIDS mortality between males and females in the 25- to 29-year-old subgroup also peaked in 2016 (RR = 2.3).

In Fig 4B, the gender disparities in the 30- to 34-year-old and 35- to 39-year-old subgroups were apparent. From 1990 to 2009, the difference between HIV/AIDS mortality among males and females in the two groups was roughly the same (with similar RRs). However, since 2009, the difference in the 35- to 39-year-old subgroup gradually became higher than that of the 30- to 34-year-old subgroup and reached its peak in 2013 (RR = 3.5).

Fig 4C had a trend similar to those shown in Fig 4D and 4E. As shown in Fig 4C, 4D and 4E, the gender differences in HIV/AIDS mortality remained significant but decreased with age. In 2016, the RRs of the 40- to 44-year-old, 45- to 49-year-old, 50- to 54-year-old, 55- to 59-year-old, 60- to 64-year -old, and 65- to 69-year-old age groups were 3.4, 3.7, 3.0, 3.2, 2.8, and 2.8, respectively. However, for the 70- to 74-year-old age group, the gender disparity began to increase with age and peaked in the 75- to 79-year-old age group (see Fig 4F).

In summary, the gender difference in HIV/AIDS mortality was readily apparent and increased gradually with age. For example, in 2016, with increasing age, the RR increased from 1.2 (15- to 19-year-old subgroup) to 5.7 (75- to 79-year-old subgroup), for a nearly five-fold total increase.

## Discussion

We found that HIV/AIDS mortality was continuously increasing in China and that gender differences were becoming more evident over time. The data of this study also correspond to those of the two periods of the development of AIDS in China. First, from 1990 to 1993, the HIV-related mortality increased from 0.33 per 100,000 people to 0.58 per 100,000 people and expanded to 21 Chinese provinces; this time interval was termed the diffusion period. Second, after 1993, the mortality of HIV/AIDS has increased rapidly, from 0.63 per 100,000 people in 1994 to 2.50 per 100,000 people in 2016, nearly 4 times the mortality in 1994. In this period, 31 provinces and municipalities in China reported HIV-infected persons; this time interval was termed the rapid growth period. In theory, with the advent of antiretroviral treatment (ART), the mortality rate would decrease significantly, which was also reported by previous studies[14,22]. However, great numbers of people who do not meet the treatment criteria may have higher mortality rates; therefore, the HIV/AIDS mortality may continue to rise from 1990 to 2016. In terms of gender differences, the 27-year period can also be divided into two stages in China. First, from 1990 to 2008, the male HIV/AIDS mortality was double the female mortality. Second, after 2008, the gap between male and female mortality became wider. By 2016, male mortality was nearly three times that of females. This result could be attributed to the pattern of the HIV epidemic, which has changed over time, and men who have sex with men (MSM) now account for more new infections than before. The contribution of our study is the finding that gender disparities in HIV/AIDS mortality became larger over time, and this study is also one of the few reports on the long-term trends of gender differences in HIV/AIDS mortality in China.

One reason why HIV/AIDS mortality among males was higher than that among females is the significant gender gap in China. According to several censuses, the sex ratios (male vs female ratios) at birth (SRB) were 111.14 in 1990, 116.86 in 2000, 117.94 in 2010 and 115.88 in 2014[23]. This difference may cause a higher HIV prevalence among males, which could then cause higher mortality among males. In addition, another Chinese survey showed that between 1990 and 2000, the male mortality was higher than the female mortality, and this difference has widened[24].

The second reason is that MSM not only have the highest HIV incidence internationally but also bear a disproportionate burden of HIV infection in China[22, 25]. Moreover, some high-risk sexual behaviors, such as inconsistent condom use or having multiple sexual partners or alcohol/drug abuse, would put MSM at a higher risk for HIV infection than normal individuals[14,26,27]. The proportion of MSM increased from 1.5% in 2006 to 23.4% in 2018, an almost 15-fold increase; that mode of transmission was the fastest-growing method for HIV/AIDS spread in China[28,29]. Therefore, the number of infected men was higher than the number of infected women in China.

The third reason for these gender differences is the outcomes of treatment for HIV/AIDS. Many studies have shown that ART not only helps to reduce the global death rate of HIV/AIDS but also benefits women more than men[10, 11]. Since the Chinese government provided ART drugs for free in 2002, the free ART database, which was established to monitor and evaluate the treatment process, suggested a positive association between the female sex and positive treatment outcomes[30,31]. Another article drew a similar conclusion regarding gender differences in the 2-year mortality of the HIV-infected Chinese population[14]. Compared with men, women not only experience a better effect of ART but also have better adherence to treatment. Therefore, more effort should be made to encourage HIV-infected males to participate in ART—the earlier, the better.

Finally, the gender differences in HIV/AIDS mortality may also be attributed to genes. Previous studies indicated that HIV-infected women tend to have higher CD4^+^ levels, meaning they may experience a more favorable course of disease than men[32]. Women also have a better response to the drugs biologically[14]. In addition, prenatal HIV testing, family planning, and gynecological services provide women with wider access to HIV testing and timely treatment[33,34]. We strongly recommend that MSM participate in counseling and testing services to verify their HIV status.

In this study, males and females in the 35- to 49-year-old age group had a higher HIV/AIDS mortality rate. The results were similar to those reported in other provinces and cities in China[35,36,37]. Most of the causes of death were AIDS-related diseases, and the survival period (from diagnosis to death) was 5 to 15 years. In addition, the elderly males (age group 65–79) had the highest mortality rate, which may be because the average life expectancy of men in China in 2015 is 73.64[38]. Most men approximately 70 years old, even if they are not HIV-infected or AIDS patients, will die of natural death or other diseases. The mortality of the 20- to 24-year-old subgroup was higher than that of the 15- to 19-year-old subgroup, and few gender differences in HIV/AIDS mortality emerged in the 15- to 19-year-old subgroup. However, for the 20- to 24-year-old subgroup, HIV/AIDS mortality among males was almost 1.5–2 times that among females. Despite the fact that some Chinese adolescents have sex at the age of 10, the average age of the onset of their sexual behavior is 18–20 years. In addition, given the long duration between initial infection with HIV/AIDS to death, some 15- to 19-year-old teenagers who contract sexually transmitted infections die at 20–24 years of age. To date, there have been few studies on HIV/AIDS among adolescents[39–42], but the impact of this epidemic on this age group cannot be underestimated, especially for young males. Considering the characteristics of the age group, adolescent (15–24 year old) males are more likely influenced by homosexuality-related discrimination and exhibit high-risk behaviors, thus leading to infection. Policymakers should focus on taking measures to prevent the spread of this epidemic among young teenagers.

The gender difference of HIV/AIDS mortality increased with age and reached the maximum in the 75- to 79-year-old age group. This phenomenon can be explained by the following four points: First, the gender gap in China is high, and the number of infected women is lower than the number of infected men in China[30]. Second, women experience a more favorable course of disease[33]. Third, women have better adherence to the ART and have better response to the drugs[14]. Last, the average life expectancy of women in China is older than that of men[38].

From this study, we also discovered a frustrating phenomenon: HIV/AIDS mortality worldwide is decreasing, while HIV/AIDS mortality in China is still steadily increasing. Because of the widespread use of ART, there was a 48% decline in the number of deaths from AIDS-related causes in the world. In eastern and southern Africa, the number of deaths from AIDS-related illnesses showed the sharpest decrease, from 1.1 million in 2004 to 420,000 in 2016[43]. Although the introduction of ART has also helped many people infected with HIV/AIDS in China, the mortality of HIV/AIDS is still on the rise[44, 45]. Previous studies have shown that highly active antiretroviral therapy (HAART), late diagnosis, poverty, lower education, and being older than 40 years of age were strongly related to AIDS-associated death[22,46]. Therefore, Chinese policymakers should expand testing to increase early HIV diagnosis and recommend ART to all diagnosed individuals as early as possible to reduce the number of AIDS-related deaths[47].

The limitations of our study are as follows. First, the data we analyzed are only speculative data of the GBD; it is possible that unintentional errors occurred when estimating the mortality rates and comparing the trends. However, the GBD 2016 collected a large sample of globally representative data, and the estimated mortality was standardized according to the 2010 age-sex distribution in China. Therefore, the conclusions drawn from the data are accurate and representative. The second limitation of our study is the age scale. The population of interest in this survey was not the whole population, as the data lack representation from the populations aged 0–10 years and over 79 years. Very few surveys have been conducted to examine HIV/AIDS mortality in these two age groups. Considering the fact that these age groups comprise only a small proportion of the population, the absence of these data in our surveys is unlikely to change the findings of this study.

Our research reveals the gender disparities in HIV/AIDS mortality. Given that this difference has increased over time, policymakers should make use of gender-specific approaches in the development of intervention strategies to reduce the mortality of HIV/AIDS in China. We propose several methods to slow the growth of HIV/AIDS mortality. First, we have promoted public HIV/AIDS education beginning in primary school to reduce the HIV/AIDS infection. Second, the groups at high risk for HIV should be regularly tested for HIV, and once these individuals become infected, they should receive ART immediately to prolong life. We also found very few studies on the complex causes of gender disparities in HIV/AIDS mortality in China; therefore, it is necessary to design cohort studies to better understand the effects of age, ART and other factors on HIV/AIDS mortality in men and women.

In conclusion, the mortality of HIV/AIDS in China is increasing, and the difference between males and females is gradually widening. Our study on this gender difference in mortality provides a basis for policymakers to formulate policies to eliminate these disparities and to ensure that everyone can live a long life in full health.

## Supporting information

S1 Table Estimated populations of different age groups in China from 1990 to 2016. The number of deaths in each age group divided by the mortality rate in each age group.
(DOCX)
