# Supplementary material for: Secular trends in HIV/AIDS mortality in China from 1990 to 2016: Gender disparities
Source: PLoS One. 2019 Jul 18;14(7):e0219689. doi: 10.1371/journal.pone.0219689 (PMC6638923; doi:10.1371/journal.pone.0219689)
Supplement: S1 Table — The number of deaths in each age group divided by the mortality rate in each age group. (DOCX) [file pone.0219689.s001.docx]

**S1 Table. Estimated Populations of Different Age Groups in China from 1990 to 2016**

| **Age**  **Group**  **(yrs)** | **10~14** | **15~19** | **20~24** | **25~29** | **30~34** | **35~39** | **40~44** | **45~49** | **50~54** | **55~59** | **60~64** | **65~69** | **70~74** | **75~79** |
| --- | --- | --- | --- | --- | --- | --- | --- | --- | --- | --- | --- | --- | --- | --- |
| **Male** | | | | | | | | | | | | | | |
| **1990** | 52874293 | 65222714 | 67584191 | 56589810 | 46110547 | 47343615 | 35261306 | 27284462 | 25302927 | 22777380 | 18258673 | 13463025 | 8748601 | 4923141 |
| **1991** | 51851955 | 62366617 | 67650362 | 61120446 | 45388134 | 49016825 | 37116524 | 28299889 | 25268371 | 23232225 | 18792831 | 13887346 | 9031991 | 5136586 |
| **1992** | 51304409 | 59381957 | 66985449 | 64833080 | 46378766 | 49650364 | 38972311 | 29682768 | 25300563 | 23607828 | 19292678 | 14315698 | 9334223 | 5366558 |
| **1993** | 51691767 | 56491938 | 65759962 | 67270771 | 49451595 | 48715393 | 41968704 | 30603603 | 25556706 | 23803826 | 19767430 | 14782293 | 9662457 | 5592322 |
| **1994** | 52968407 | 54103168 | 63985117 | 68573882 | 53723418 | 46887961 | 43759983 | 33363354 | 25750761 | 23813151 | 20332041 | 15271584 | 10083297 | 5754659 |
| **1995** | 54954535 | 52529583 | 61718032 | 68902281 | 58303415 | 45229312 | 46588519 | 34848332 | 26137274 | 23823053 | 20811941 | 15774554 | 10493259 | 5974989 |
| **1996** | 58185640 | 51504930 | 59094326 | 68541562 | 62358815 | 44726247 | 48038427 | 36622021 | 27180332 | 23801723 | 21265691 | 16250068 | 10882706 | 6214362 |
| **1997** | 62230051 | 50974911 | 56317790 | 67660251 | 65461104 | 45948335 | 48495277 | 38360601 | 28597123 | 23841109 | 21671534 | 16696441 | 11296747 | 6469036 |
| **1998** | 65436785 | 51418465 | 53601041 | 66380197 | 67250926 | 49228026 | 47461240 | 41192282 | 29561299 | 24094448 | 21933326 | 17120829 | 11758746 | 6732137 |
| **1999** | 67265595 | 52804309 | 51340481 | 64651952 | 67982093 | 53642527 | 45609413 | 42824730 | 32308407 | 24296977 | 22042521 | 17624930 | 12250110 | 7050015 |
| **2000** | 67686544 | 54967962 | 49850136 | 62466350 | 67935452 | 58263452 | 43974049 | 45479265 | 33780436 | 24690899 | 22152693 | 18070910 | 12753591 | 7377930 |
| **2001** | 65895790 | 58463716 | 48889674 | 59897275 | 67504057 | 62230454 | 43512265 | 46822444 | 35506884 | 25715659 | 22221392 | 18530111 | 13220760 | 7716843 |
| **2002** | 62350132 | 62807137 | 48405691 | 57150240 | 66762441 | 65203129 | 44745199 | 47241233 | 37177554 | 27086382 | 22334142 | 18969357 | 13654349 | 8081864 |
| **2003** | 58518541 | 66285823 | 48854341 | 54441640 | 65738494 | 66892218 | 47975311 | 46234304 | 39890956 | 28012040 | 22631220 | 19275707 | 14065432 | 8469786 |
| **2004** | 55033274 | 68331268 | 50204723 | 52168990 | 64299713 | 67590880 | 52286583 | 44439260 | 41467466 | 30628647 | 22866423 | 19441583 | 14548202 | 8870403 |
| **2005** | 51890590 | 68929552 | 52304079 | 50643435 | 62379618 | 67582548 | 56766978 | 42861351 | 44025694 | 32027444 | 23276661 | 19621607 | 14997178 | 9298233 |
| **2006** | 49300365 | 67322230 | 55703282 | 49618845 | 60032476 | 67222931 | 60587720 | 42439452 | 45316902 | 33682888 | 24287014 | 19796439 | 15468577 | 9733064 |
| **2007** | 47169010 | 63948044 | 59948104 | 49045078 | 57460108 | 66570042 | 63428720 | 43678177 | 45709527 | 35293751 | 25632816 | 20028654 | 15933071 | 10167096 |
| **2008** | 45204639 | 60243354 | 63388025 | 49377693 | 54870613 | 65636449 | 65026889 | 46854908 | 44731618 | 37907037 | 26551479 | 20415468 | 16292079 | 10592953 |
| **2009** | 43336529 | 56817406 | 65485819 | 50607011 | 52664932 | 64281634 | 65691307 | 51070416 | 43013106 | 39455596 | 29066046 | 20715651 | 16538601 | 11070131 |
| **2010** | 41829387 | 53642270 | 66238357 | 52603416 | 51164741 | 62429533 | 65710498 | 55444847 | 41520847 | 41954743 | 30406809 | 21140143 | 16780397 | 11497416 |
| **2011** | 40825441 | 50900816 | 64893648 | 55927867 | 50121514 | 60119125 | 65427127 | 59174727 | 41144980 | 43250955 | 31992068 | 22075316 | 16986381 | 11912789 |
| **2012** | 40059028 | 48581536 | 61827288 | 60128219 | 49509891 | 57559413 | 64876473 | 61958013 | 42365272 | 43684478 | 33540822 | 23286086 | 17215227 | 12308109 |
| **2013** | 39676079 | 46443105 | 58406378 | 63561747 | 49808179 | 54971380 | 64056920 | 63544782 | 45453124 | 42806890 | 36032443 | 24083267 | 17568197 | 12633264 |
| **2014** | 39657450 | 44438751 | 55209975 | 65676135 | 51009757 | 52761513 | 62816847 | 64230705 | 49531072 | 41218991 | 37526906 | 26328967 | 17834183 | 12896518 |
| **2015** | 39650013 | 42835521 | 52213208 | 66446032 | 52981908 | 51251722 | 61075634 | 64290938 | 53736738 | 39839290 | 39882123 | 27440638 | 18181470 | 13156753 |
| **2016** | 39628855 | 41752918 | 49617223 | 65105775 | 56287718 | 50194758 | 58875496 | 64058563 | 57307343 | 39516671 | 41103470 | 28783291 | 18947243 | 13380519 |
| **Total** | **1396475104** | **1513510001** | **1565476702** | **1619389980** | **1542940425** | **1521149786** | **1420105720** | **1232905427** | **1012643282** | **843864081** | **695673193** | **523189963** | **374527075** | **238377476** |
| **Female** | | | | | | | | | | | | | | |
| **1990** | 49556830 | 61797527 | 64611324 | 53588740 | 42251745 | 44190534 | 31979605 | 24420999 | 22494590 | 20679693 | 17187401 | 13905441 | 10113145 | 6498445 |
| **1991** | 48579956 | 59207838 | 64032115 | 57892695 | 41781897 | 45516419 | 33909557 | 25510785 | 22525468 | 21104613 | 17612996 | 14218963 | 10365412 | 6724791 |
| **1992** | 48056919 | 56459771 | 62935729 | 61312924 | 42939148 | 45866245 | 35932605 | 26839988 | 22630345 | 21487216 | 17968396 | 14563778 | 10629832 | 6959865 |
| **1993** | 48369118 | 53737316 | 61617545 | 63426342 | 45999375 | 44791426 | 39004756 | 27860012 | 22835483 | 21690689 | 18328769 | 14908469 | 10949064 | 7197527 |
| **1994** | 49425600 | 51441818 | 60087710 | 64377271 | 50157613 | 43018606 | 41161596 | 30175850 | 23244997 | 21489553 | 18909343 | 15290239 | 11306548 | 7360398 |
| **1995** | 51079102 | 49930015 | 58281853 | 64371136 | 54637095 | 41552989 | 43571058 | 31853413 | 23755660 | 21538509 | 19352717 | 15672324 | 11638185 | 7570975 |
| **1996** | 53872673 | 49028434 | 56175463 | 63735001 | 58695926 | 41275341 | 44698599 | 33726177 | 24880366 | 21578098 | 19769556 | 16054111 | 11949387 | 7803328 |
| **1997** | 57416788 | 48583394 | 53913292 | 62839846 | 61820273 | 42656977 | 44899719 | 35661632 | 26251655 | 21690071 | 20170824 | 16368051 | 12299768 | 8052643 |
| **1998** | 60241762 | 49004575 | 51638667 | 61894613 | 63644957 | 45945929 | 43742571 | 38612622 | 27314164 | 21901785 | 20422458 | 16694231 | 12659854 | 8351225 |
| **1999** | 61846986 | 50234424 | 49716096 | 60811945 | 64374102 | 50308686 | 41949796 | 40642292 | 29650710 | 22315506 | 20306609 | 17237251 | 13052601 | 8679857 |
| **2000** | 62139542 | 52169377 | 48500409 | 59416662 | 64267041 | 54928856 | 40512757 | 42937663 | 31334518 | 22830906 | 20430442 | 17670078 | 13438972 | 8986923 |
| **2001** | 60293200 | 55423675 | 47851031 | 57570997 | 63690896 | 59016324 | 40293332 | 44002990 | 33188792 | 23947302 | 20539890 | 18099395 | 13819392 | 9279037 |
| **2002** | 56757768 | 59530760 | 47606061 | 55411093 | 62926905 | 62104776 | 41719342 | 44194044 | 35090415 | 25302535 | 20710119 | 18518980 | 14149028 | 9608262 |
| **2003** | 52882911 | 62896661 | 48175228 | 53105533 | 62101503 | 63871002 | 45009913 | 43081408 | 37981825 | 26347304 | 20964999 | 18792162 | 14492705 | 9948908 |
| **2004** | 49307266 | 64950396 | 49514059 | 51064581 | 61062225 | 64542617 | 49328962 | 41357794 | 39985561 | 28621697 | 21406305 | 18725368 | 15025892 | 10317415 |
| **2005** | 46075113 | 65587057 | 51536893 | 49685910 | 59610349 | 64384815 | 53867707 | 39983407 | 42250055 | 30261590 | 21947437 | 18890771 | 15458463 | 10690985 |
| **2006** | 43410484 | 63972253 | 54875873 | 48852425 | 57638167 | 63759734 | 57862894 | 39805319 | 43308890 | 32076003 | 23065473 | 19063756 | 15892378 | 11079706 |
| **2007** | 41236943 | 60538456 | 59086600 | 48419568 | 55329596 | 62952775 | 60866499 | 41251472 | 43507240 | 33941763 | 24414535 | 19312175 | 16326771 | 11448372 |
| **2008** | 39268815 | 56657642 | 62572237 | 48813689 | 52878079 | 62087359 | 62571702 | 44537571 | 42424792 | 36776992 | 25464633 | 19641259 | 16642868 | 11833225 |
| **2009** | 37442710 | 52976954 | 64756218 | 50008105 | 50712521 | 61011692 | 63216943 | 48836124 | 40750037 | 38768081 | 27706239 | 20130180 | 16668176 | 12367423 |
| **2010** | 35992238 | 49528726 | 65530955 | 51930101 | 49243913 | 59526754 | 63073239 | 53347740 | 39426689 | 41022042 | 29332002 | 20698895 | 16896211 | 12801105 |
| **2011** | 35047526 | 46537154 | 64045077 | 55215298 | 48361704 | 57521560 | 62500661 | 57313736 | 39280951 | 42109201 | 31126493 | 21793038 | 17113694 | 13212632 |
| **2012** | 34330924 | 44021436 | 60707961 | 59402873 | 47911636 | 55189594 | 61767157 | 60297164 | 40731025 | 42356324 | 32969746 | 23082640 | 17377781 | 13599064 |
| **2013** | 33946118 | 41738797 | 56884706 | 62884078 | 48307431 | 52730601 | 60981754 | 61999160 | 43988154 | 41354576 | 35748277 | 24063656 | 17699015 | 13880688 |
| **2014** | 33859405 | 39648570 | 53222786 | 65067813 | 49508959 | 50570990 | 59985306 | 62656477 | 48230428 | 39772130 | 37711117 | 26166003 | 18149723 | 13927212 |
| **2015** | 33761191 | 37994732 | 49763263 | 65834832 | 51433691 | 49113481 | 58578171 | 62536328 | 52666181 | 38523589 | 39915106 | 27656985 | 18662129 | 14145614 |
| **2016** | 33625292 | 36889170 | 46751264 | 64331966 | 54707007 | 48238419 | 56651167 | 61991692 | 56558938 | 38414235 | 40989357 | 29324469 | 19643907 | 14361623 |
| **Total** | **1257823180** | **1420486928** | **1514390415** | **1561266037** | **1465993754** | **1436674501** | **1339637368** | **1165433859** | **956287929** | **797902003** | **664471239** | **516542668** | **392420901** | **276687248** |
